# Development of Antibody–Drug Conjugates Using DDS and Molecular Imaging

**DOI:** 10.3390/bioengineering4030078

**Published:** 2017-09-17

**Authors:** Masahiro Yasunaga, Shino Manabe, Atsushi Tsuji, Masaru Furuta, Koretsugu Ogata, Yoshikatsu Koga, Tsuneo Saga, Yasuhiro Matsumura

**Affiliations:** 1 Division of Developmental Therapeutics, EPOC, National Cancer Center, Kashiwa 277-8577, Japan; ykoga@east.ncc.go.jp (Y.K.); yhmatsum@east.ncc.go.jp (Y.M.); 2 Synthetic Cellular Chemistry Laboratory, RIKEN, Wako 351-0198, Japan; smanabe@riken.jp; 3 Department of Molecular Imaging and Theranostics, National Institute of Radiological Sciences, QST, Chiba 263-8555, Japan; abtsuji+nirs@gmail.com; 4 Shimadzu Corporation, Kyoto 604-8511, Japan; furu@shimadzu.co.jp (M.F.); kogata@shimadzu.co.jp (K.O.); 5 Department of Diagnostic Radiology, Kyoto University Hospital; Kyoto 606-8501, Japan; saga@kuhp.kyoto-u.ac.jp

**Keywords:** ADC (antibody-drug conjugate), DDS (drug delivery system), molecular imaging, antibody delivery, controlled release, PET (positron emission tomography), MSI (mass spectrometry imaging)

## Abstract

Antibody-drug conjugate (ADC), as a next generation of antibody therapeutics, is a combination of an antibody and a drug connected via a specialized linker. ADC has four action steps: systemic circulation, the enhanced permeability and retention (EPR) effect, penetration within the tumor tissue, and action on cells, such as through drug delivery system (DDS) drugs. An antibody with a size of about 10 nm has the same capacity for passive targeting as some DDS carriers, depending on the EPR effect. In addition, some antibodies are capable of active targeting. A linker is stable in the bloodstream but should release drugs efficiently in the tumor cells or their microenvironment. Thus, the linker technology is actually a typical controlled release technology in DDS. Here, we focused on molecular imaging. Fluorescent and positron emission tomography (PET) imaging is useful for the visualization and evaluation of antibody delivery in terms of passive and active targeting in the systemic circulation and in tumors. To evaluate the controlled release of the ADC in the targeted area, a mass spectrometry imaging (MSI) with a mass microscope, to visualize the drug released from ADC, was used. As a result, we succeeded in confirming the significant anti-tumor activity of anti-fibrin, or anti-tissue factor-ADC, in preclinical settings by using DDS and molecular imaging.

## 1. Introduction

Antibody-drug conjugate (ADC) is a next generation therapeutic antibody. Several ADCs have been used in clinics already [1,2,3,4]. Moreover, a large number of biotech and pharmaceutical companies are dealing with ADC and are competitively exploiting new ones [5,6]. Over 40 ADCs are under clinical trials worldwide [1,2,7]. However, the effectiveness of ADCs in treating relapsed or refractory malignant diseases is their most important aspect. SGN-35 is effective for patients with CD30-positive relapsed or refractory malignant lymphoma [8,9,10]. T-DM1 is also effective for patients with HER2-positive advanced or remnant breast cancer previously treated with standard dugs, including the naked anti-HER2 antibody [10,11]. Hence, ADC has been expected to be a breakthrough drug following the immune checkpoint blockades.

**Figure 1:**
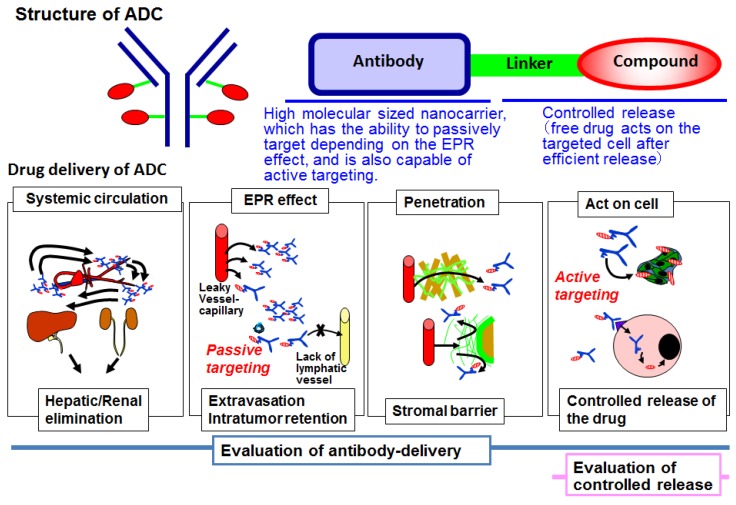
Structure and drug delivery of antibody-drug conjugate (ADC). ADC has three parts: antibody, linker, and drug. ADC has four action steps: systemic circulation, enhanced permeability and retention (EPR) effect, penetration, and action on cells, like drug delivery system (DDS) drugs.

ADC has three parts: antibody, linker, and drug. An antibody is a large molecular-sized carrier, which has the ability for passive targeting depending on the enhanced permeability and retention (EPR) effect [12,13]. It is also capable of active targeting depending on the specific recognition and binding to the target antigen [5,14]. A linker is stable in the bloodstream but should efficiently release the drug in the tumor cells or within their microenvironment [2,6,15]. The total number of drugs conjugated with a single antibody molecule is about four, but can be up to eight. Therefore, highly toxic agents are strongly required [1,2,6,15]. Pharmacologically, ADC has four action steps: systemic circulation, the EPR effect including passive targeting, penetration within the tumor tissue, and action on cells, which includes active targeting and controlled release. This mechanism is similar to drug delivery system (DDS) drugs, such as liposome or micelle (Figure 1). The linker technology is a typical controlled release technology in DDS. It is clear that ADC should belong to the DDS drug category. Here, we focused on molecular imaging which helps visualize the antibody delivery throughout the four steps, including the controlled release in the final step. Here, we review the development of ADC and our recent research works using DDS and molecular imaging.

## 2. Antibody–Drug Conjugate

ADC technologies have been developed for the targeted delivery of agents while minimizing their adverse effects. First-generation ADCs were produced with murine-derived antibody backbones. Therefore, an anti-mouse antibody generated in the human body (HAMA, human anti-mouse antibody) accelerated the clearance of ADCs by host immune reaction. The linkers were not stable enough in the bloodstream. Collectively, ADCs themselves showed a short half-life in the human body. Moreover, the drugs used as a payload (IC_50_, half maximal (50%) inhibitory concentration; μM level) were not toxic enough to be significantly effective in human subjects. Consequently, ADCs dropped out of clinical trials. In addition, the FDA-approved Gemtuzumab ozogamicin was also withdrawn from the market because of serious toxicities. However, recent advances in bioengineering have improved these drawbacks, resulting in the emergence of second generation ADCs. Since then, many methods have been used to improve both the stability in the bloodstream and the controlled drug release in the targets, which has led to demonstrating clinical effectiveness, including SGN-35, anti-CD30 chimeric antibody (human constant regions with down-sized mouse variable regions) with monomethyl auristatin E (MMAE, IC_50_; nM level) via valine-citrulline (cathepsin cleavable) linker and T-DM1, anti-HER2 humanized antibody (largely human component with minimized mouse CDR segment) with Maytansine (IC50; nM level) via a thioester (noncleavable) linker, which have lower immunogenicity [1,2,5,10].

The heterogeneity of the drug-antibody ratio (DAR), which is the number of drug molecules loaded onto single antibody, is an important issue in the development of the third generation ADCs. DAR can accelerate the clearance and weaken the efficacy of ADCs. To produce homogeneous ADCs, site-specific drug conjugation methods have been developed, including THIOMAB as a cysteine replacement strategy, or SMARTag^TM^, SMAC-TAG^TM^, and TG-ADC^TM^ as chemo-enzymatic strategies [10]. In addition, novel technologies, such as bispecific antibodies or bispecific T-cell engager (BiTE), have been applied for increasing efficacy. Furthermore, a novel combination approach with immune checkpoint blockades, or an application of immune-oncology agents as a payload, would be promising for achieving a durable response in clinics [10,16,17].

Thus, an evaluation and modification of antibody delivery and controlled drug release is important for ADC development. 

## 3. Antibody Delivery and the EPR Effect

**Figure 2:**
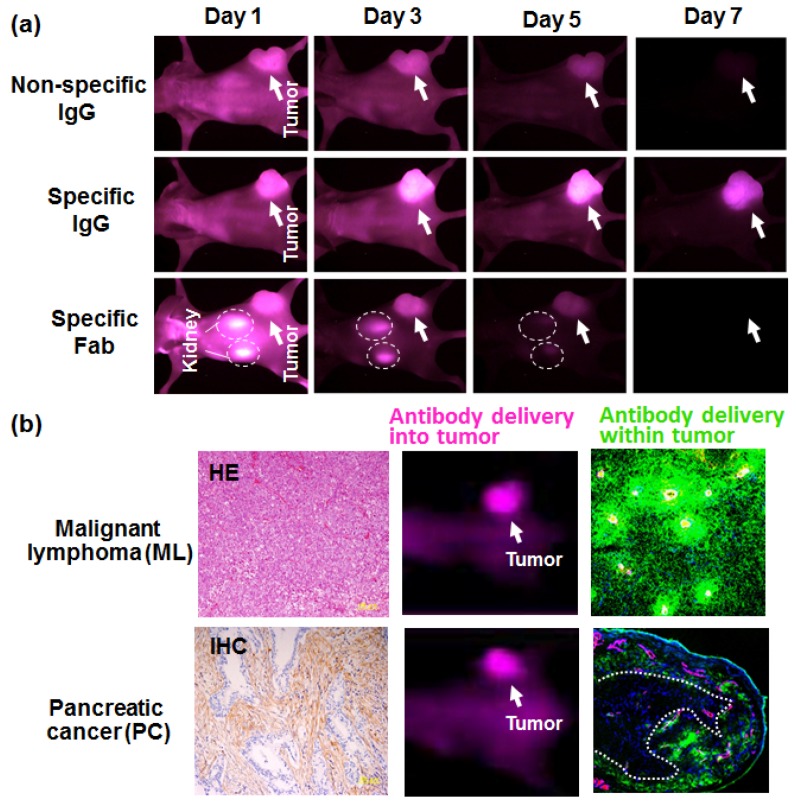
Fluorescent imaging of antibody delivery. (**a**) An in vivo imaging analysis of a mouse xenograft model was conducted on fluorescent non-specific IgG, specific IgG, or specific Fab on days one, three, five, and seven after the administration. (**b**) Left panel, hematoxylin-eosin straining of malignant lymphoma (ML) (upper panel) and immunostaining of pancreatic cancer (PC) (lower panel) in which cancer cells (blue) were surrounded by dense stromal collagen 4 (brown). The middle panel shows the in vivo imaging of fluorescent anti-CD 20 and anti-EpCAM antibody that were injected into the ML and PC model, respectively. The right panel shows the distribution of anti-CD 20 and anti-EpCAM antibody (both green) within a ML tumor and PC tumor. The blood vessels, yellow in the upper panel and magenta in the lower panel, were also observed.

High molecular weight (HMW) agents, in the range of about 10–200 nm can extravasate easily from leaky tumor vessels due to the immature structure with intercellular openings and the increased vascular permeability factor, such as VEGF or Kallikrein-Kinin [12,13,18]. In addition, because of the lack of lymphatic vessels acting as a drainage system for HMW agents, they can stay in the tumor for a long time. This mechanism is specific for cancer and is called the EPR effect [12,13,19]. An antibody, typically IgG with a size of about 10 nm acting as a HMW agent, can selectively accumulate in the tumor even if it is a non-specific antibody. This is the passive targeting of an antibody, and the mechanism depends on the EPR effect [13,19,20]. Moreover, a specific antibody can accumulate more and stay longer in the tumor than a non-specific antibody, which is called active targeting [13,19,20]. Interestingly, small-sized IgG fragments, such as Fab, accumulate in the tumor but stay less time than the specific antibody IgG, because the passive targeting ability has been lost, as seen in small compounds such as a low molecular weight (LMW) agent. Small-sized IgG fragments are also eliminated from the kidney. The absence of passive targeting and rapid renal clearance lead a loss of the long-term accumulation seen in its IgG counterpart. Finally, by using in vivo imaging, we found that specific Fab showed the same tumor accumulation as non-specific IgG (Figure 2a). These results indicated the importance of molecular imaging for observing antibody delivery in vivo. 

## 4. Antibody Delivery and Tissue Penetration

The prognosis for brain tumor glioblastoma (GBM) and pancreatic cancer (PC) remains quite poor [21,22,23,24]. GBM also involves a blood brain tumor barrier (BBTB) [22,23]. PC involves hypovascularity and a low blood supply [21]. These are all disadvantages for drug delivery. In addition, within tumor tissues, dense tumor stroma can block the penetration of the drugs [19,25,26,27,28], so most drugs cannot reach the cancer cells. This is called the stromal barrier [25,26,27,28]. In order to visualize the stromal barrier, we conducted in vivo imaging using fluorescent antibodies. Two types of models including malignant lymphoma (ML) that has less stroma and PC with dense stroma, were prepared and treated with cancer-specific anti-CD20 or the anti-EpCAM antibody, respectively [27]. Large amounts of accumulation, caused by both passive targeting and active targeting, were observed in both tumor models at seven days after the administration. Macroscopically, antibody delivery appeared successful in both tumor models. Microscopically, in the ML tumor, anti-CD20 antibody penetrated deeply into the whole tumor area, showing good distribution. On the other hand, distribution of the anti-EpCAM antibody was restricted to the peripheral area neighboring the tumor vessels. There was no clear signal in the central area. The penetration of the antibody was clearly inhibited by the tumor stroma (Figure 2b). 

To overcome this drawback, we developed anti-fibrin ADC [29] and anti-tissue factor (TF) ADC [30]. The former was named by cancer stromal targeting (CAST) therapy [13,31]. In conventional ADC, the target is the cell-surface protein of cancer cells and an intracellular drug-release type linker is used. After the internalization, the drug can be released. On the other hand, in CAST-ADC, the target is the tumor stroma and not the cell, and an extracellular drug-release type linker is used. The drug can be released outside of the cells, just on the stroma without internalization [26,27,29,31]. For the anti-TF-ADC, an anti-TF antibody binds strongly to both the tumor and stromal cells expressing TF, and can be used for simultaneous targeting of tumor and stromal cells compared to a typical CAST-ADC.

## 5. Immuno-PET Imaging

**Figure 3:**
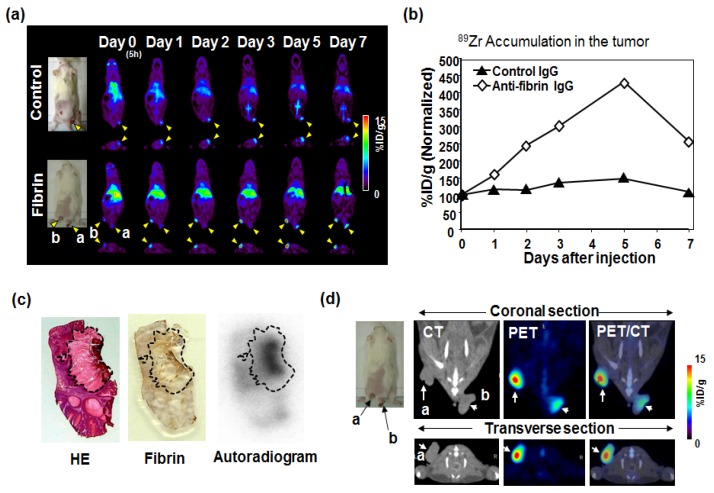
Evaluation of antibody delivery with positron emission tomography (PET) imaging. (**a**)–(**b**) PET imaging analysis was conducted using an ^89^Zr-labeled anti-fibrin antibody on day zero, one, two, three, five, and seven after the administration and %ID/g showed the relative value of Day 0 (100%). (**c**) With autoradiogram examination, the ^89^Zr-labeled anti-fibrin antibody was accumulated within the fibrin-positive tumor stroma, as represented by the dashed black line. (**d**) In PET/CT, the ^89^Zr-labeled anti-fibrin antibody showed clear and specific accumulation in the tumor. Adapted from Hisada et al. [32].

We used a positron emission tomography (PET) system to evaluate the antibody delivery [32]. The antibody was labeled with a positron-emitting radionuclide as a tracer. The PET system can enable visualization of the antibody delivery from pairs of gamma rays emitted indirectly by the labeled tracer [33]. This immuno-PET is better than fluorescent imaging for deep tissue imaging because of its high sensitivity and accurate quantification. ^64^Cu with a half-life of 13 hours, ^76^Br with a half-life of 16 hours, or ^89^Zr with a half-life of 72 hours, all having a relatively long physical half-life, are usually used for immuno-PET imaging [33,34,35]. Among them, ^89^Zr, which has a half-life of about three days, is the most commonly used for labeling antibodies worldwide, because the antibody also has long half-life of about three to seven days, and three days are required to acquire good contrast imaging after administration [34,36,37]. We conducted immuno-PET imaging of the ^89^Zr-labeled anti-fibrin antibody in a chemically-induced mouse skin cancer model similar to human skin cancer [32]. The anti-fibrin antibody selectively accumulated in the tumor. Serial PET imaging clearly showed skin cancers with ^89^Zr-labeled anti-fibrin antibody (Figure 3a). Quantification of the PET images indicated that the accumulation of the ^89^Zr-labeled anti-fibrin antibody in the tumor increased with time and peaked on day five after administration, while the control antibody did not show a time-dependent increase in tumor uptake (Figure 3b). Comparison of fibrin immunostaining and autoradiography confirmed the selective localization of the ^89^Zr-labeled anti-fibrin antibody in the fibrin-positive tumor stroma (Figure 3c). Furthermore, we used PET/CT imaging which clearly indicated that the area showing high uptake of anti-fibrin antibody coincided with the tumor area, detected by CT scan (Figure 3d). We were able to confirm the effective delivery and tumor specificity of the anti-fibrin antibody. Lastly, we succeeded in confirming the significant anti-tumor activity of anti-fibrin CAST-ADC in a preclinical setting. 

More recently, PET/MRI has been used for molecular imaging [38]. Hybrid imaging, combining functional information from PET with morphological information by CT/MRI, is expected to improve diagnostic ability and contribute to the better management of cancer patients. Although ^18^F-FDG and ^11^C-methionine, as part of a PET probe for targeting cellular metabolism, have been widely used in clinics, the use of a radionuclide-labeled antibody as a targeting PET probe has been progressively increasing in clinics [33,36,39,40]. In addition, many therapeutic antibodies, including ADC, will be increasingly used around the world. Using a companion diagnostic for the determination of the indicated treatment (e.g., the patient with HER2 positive breast cancer for T-DM1 treatment) is important [36,39,41,42]. In general, although an immunohistochemistry assay is used for the companion diagnosis, a biopsy specimen is needed. However, obtaining a biopsy from a patient with metastasis in deep organs, such as the brain or bone, is difficult [36,43]. Therefore, immuno-PET could be applied in those cases. Moreover, it can provide a non-invasive test instead of an invasive surgical biopsy. Therefore, immuno-PET would be useful for precision medicine as well as ADC development [33,36].

## 6. Mass Spectrometry Imaging

Mass spectrometry imaging (MSI) is a method to view a biomolecule or metabolite in a tissue sample by using mass spectrometry [44,45,46,47,48,49]. Ionization of the targeted molecules is important for the mass analysis. For the ionization, several methods exist, such as Matrix-Associated Laser Desorption/Ionization (MALDI) or Electrospray Ionization (ESI) [44,45,46]. ESI is capable of ionizing a wide range of molecules, including chemical compounds without the addition of a matrix under ambient conditions. The analysis can be performed in conjunction with liquid chromatography with mass spectrometry (LC-MS). 

For MALDI, the matrix should be sprayed on the tissue sample. After the laser irradiates the treated sample, ion exchange occurs between the ionized matrixes transfer protons and the analyte molecules (biomolecules and metabolites). Finally, the molecules become ionized. Mass analysis of the ionized molecules can be conducted by using Time-Of-Flight MS (TOF-MS) [44,45]. TOF-MS uses a simple principle to calculate the molecule size according to the difference in the flight time. Lighter ions of the same charge reach higher speeds, whereas heavier ones are slower. Therefore, the mass-to-charge ratio (*m/z*) of each ion can be determined by measuring the velocity. If we selected the molecule with specialized *m/z*, we can identify the molecule itself and semi-quantify it in the examination area. 

Briefly, MALDI imaging provides an enormous amount of information on the abundance and distribution of the targeted molecules within tissue samples with high sensitivity and high spatial resolution [44,45]. A suitable matrix should be selected for an efficient ionization, although it is difficult for some molecules to achieve it. On the other hand, ESI-MSI allows direct tissue analysis without matrix-preparation [46,48,49]. As a drawback, the spatial resolution is poor when compared with MALDI-MSI.

**Figure 4:**
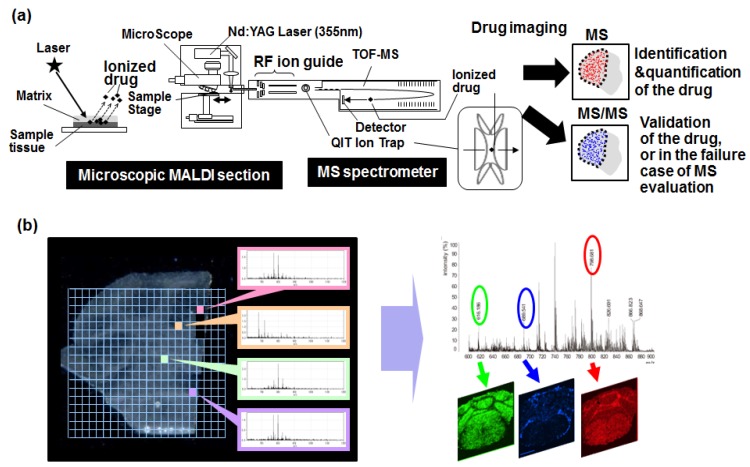
Mass spectrometry imaging (MSI) with a mass microscope. (**a**) A schematic representation of our drug imaging system using MSI with a mass microscope. (**b**) A mass microscope demonstrates the tissue distribution of targeted molecules with a high spatial resolution. Adapted from Yasunaga et al. [50].

The new MSI analyzer, namely the mass microscope, is a microscope coupled with a high-resolution atmospheric pressure-laser desorption/ionization and quadruple ion trap TOF analyzer, has improved the tissue resolution of MALDI-MSI [50,51,52,53,54]. It has a resolution of 10 μm or less, which is advantageous for evaluating the drug distribution in specific cells or areas of interest within tissues. The mass microscope also allows an image from MSI to be overlaid on an optical image in the same sample, which is beneficial for understanding and analyzing the tissues of interest. Here, we hypothesized that MSI using a mass microscope should be able to be applied for the evaluation of the controlled release of ADC in the targeted area (Figure 4a,b) [50].

## 7. Visualization of the Controlled Release

**Figure 5:**
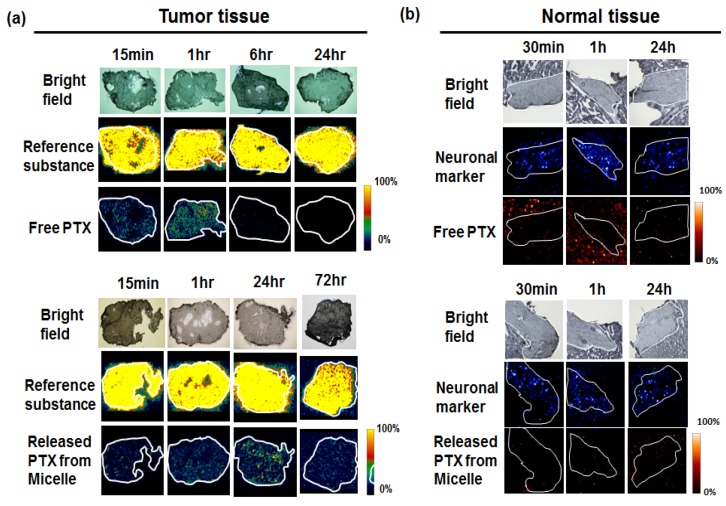
Visualization of the controlled release of PTX-incorporated micelle. (**a**) In tumor tissue, the bright field (upper), reference substance (middle, an arbitrary signal of 824.6 *m/z*), and PTX (lower, specific signal of 892.3 *m/z*). (**b**) In normal tissue, bright field (upper), neuronal marker (middle, sphingomyelin-specific signal of 851.6 *m/z*), and PTX (lower, specific signal of 892.3 *m/z*). The neuronal area is delineated by a white line. Adapted from Yasunaga et al. [50].

Before the MSI examination, we thought it might be difficult to detect the drug signal because of the limitation on the sensitivity. A single ADC only has about four drug molecules, or eight at most. Our concern was that the concentration of the released drug might be below the detection limit. We decided to evaluate the paclitaxel (PTX)-incorporated micelle [13,55] as a DDS drug, which initially contained about 200 PTX molecules. Free PTX (fPTX), as a conventional control and released PTX (rPTX) from micelles, were detected in the tumor. A fPTX signal was detected at 15 min and one hour after the administration, but decreased at six hours and disappeared by 24 hours. In contrast to fPTX, a rPTX signal was detected from 15 min to 72 hours after administration. The signal intensity was greatest at 24 hours (Figure 5a). Next, we conducted drug imaging in normal neuronal tissue. A strong fPTX signal was detected in the perineuronal lesion at 30 min and one hour after the administration. By contrast, the rPTX signal from PTX-micelle was extremely weak around the neuron (Figure 5b). This is a significant difference and the reason why PTX-micelle does not cause neurotoxicity [13]. We thus succeeded in visualizing the EPR effect for the first time [50].

**Figure 6:**
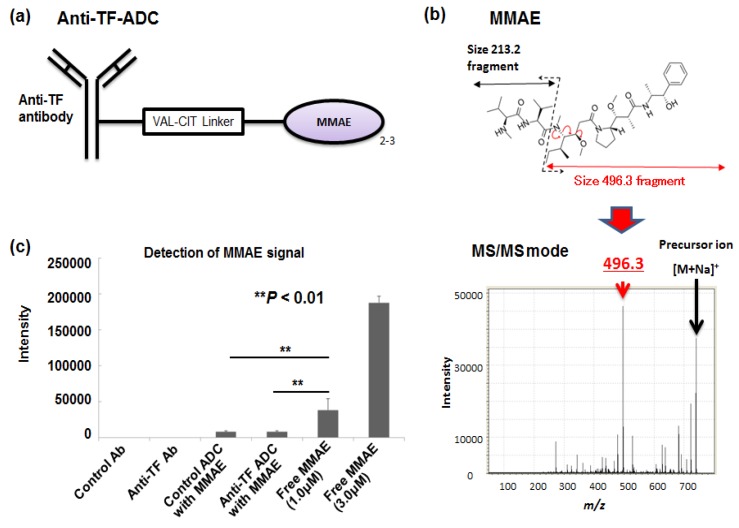
Visualization of released monomethyl auristatin E (MMAE) from ADC. (**a**) Drug design of the anti-tissue factor (TF) antibody-drug conjugate (anti-TF-ADC). (**b**) The MMAE-specific fragment with a size of 496.3 *m/z* was determined in the MS/MS analysis. (**c**) In MSI analysis, released MMAE (MMAE alone) was clearly distinguished from MMAE conjugated in ADC (ADC with MMAE). Adapted from Fujiwara et al. [51].

**Figure 7:**
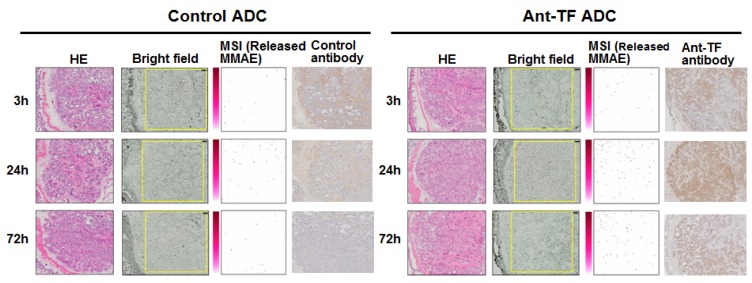
Evaluation of the controlled release of MMAE from ADC using MSI. Tumor samples from the mouse xenograft model were prepared on three, 24, and 72 h after the administration of the control ADC and anti-TF-ADC, respectively. In each ADC, H&E staining (far left) and bright field (left-middle) are shown. The rectangles on the bright field show the measurement area. The released MMAE signals obtained from 496.3 *m/z* using a mass microscope is shown. The signals of antibody/ADC were acquired from immunostaining with horseradish peroxidase (HRP) labelled each antibody. Adapted from Fujiwara et al. [51].

Subsequently, we wanted to visualize the anti-tissue factor antibody-drug conjugate (anti-TF-ADC) (Figure 6a) [51]. Monomethyl auristatin E (MMAE) was used as a payload [1,2,3,8,9]. The molecular weight (MW) of MMAE is 717.5. The three positive-ion peaks are derived from MMAE: 718.4, 740.4, and 756.4 *m/z* as a single-charge hydrogen [M + H]+, sodium [M + Na]+, and potassium [M + K]+, respectively, were observed by MS analysis. We then examined the MS/MS fragments of MMAE from each of the three positive-ion peaks. Among them, we selected the prominent fragment 496.3 *m/z* detected when 740.4 *m/z* was used as a precursor ion. In the validation tests, the specificity of 496.3 *m/z*, as a MMAE-specific fragment peak, was confirmed. We thus succeeded in visualizing and quantifying MMAE separately from other biomolecules (Figure 6b) [51]. In an in vitro study, the intensities of the mAbs, ADCs, and each MMAE sample were measured. The signal intensity of free MMAE increased in a concentration-dependent manner. Moreover, the signal intensities obtained from 1.0 μL of 1.0-μM human TF ADC and the control ADC were far weaker than those from 1.0 μL of 1.0-μM MMAE alone. Therefore, the MMAE signal in tumor tissues, after the ADC treatment, was largely released MMAE. These observations were able to be performed in a stable and reproducible manner, with a high-resolution atmospheric pressure mass microscope (Figure 6c). We then concluded that the controlled release of ADC can be visualized and quantified by MSI. Control ADC or anti-TF-ADC was administered into a mouse bearing a human pancreatic cancer tumor. In the examination of the tumor samples by MSI, a strong released MMAE signal from anti-TF-ADC was detected when compared to that of the control-ADC. The signal was strongest at 24 h after the administration. The data was validated by LC-MS analysis [51]. We concluded that ADC distribution and controlled drug release were successful in the tumor area (Figure 7) [51]. In accordance with these results, a significant anti-tumor effect of anti-TF ADC has been recognized in the xenograft model of PC [30].

## 8. Conclusions

We described our recent work in the development of ADCs as follows. 1) ADC, as a next generation of antibody therapeutics, has been expected to be a breakthrough drug following the immune checkpoint blockades. 2) ADC has four action steps: systemic circulation, the EPR effect which is passive targeting, penetration within the tumor tissue, and action on cells, which involves the active targeting and controlled release, like DDS drugs. Therefore, the evaluation of both antibody delivery and controlled release is important. 3) Fluorescent/PET imaging and MSI are useful for the evaluation of antibody delivery and controlled release, respectively, in ADC research, development, and medicine. 4) We successfully developed novel ADCs, anti-fibrin-ADC, anti-tissue factor (TF)-ADC, and others [26,27,29,30,31,56] by using DDS and molecular imaging.
